# TREM2 is down-regulated by HSV1 in microglia and involved in antiviral defense in the brain

**DOI:** 10.1126/sciadv.adf5808

**Published:** 2023-08-18

**Authors:** Stefanie Fruhwürth, Line S. Reinert, Carl Öberg, Marcelina Sakr, Marcus Henricsson, Henrik Zetterberg, Søren R. Paludan

**Affiliations:** ^1^ Department of Rheumatology and Inflammatory Research, Institute of Medicine, Sahlgrenska Academy at the University of Gothenburg, Gothenburg, Sweden.; ^2^ Department of Psychiatry and Neurochemistry, Institute of Neuroscience and Physiology, Sahlgrenska Academy at the University of Gothenburg, Gothenburg, Sweden.; ^3^ Department of Biomedicine, Aarhus University, Aarhus, Denmark.; ^4^ Biomarker Discovery and Development, Research and Early Development, Cardiovascular, Renal, and Metabolism (CVRM), BioPharmaceuticals R&D, AstraZeneca, Gothenburg, Sweden.; ^5^ Clinical Neurochemistry Laboratory, Sahlgrenska University Hospital, Mölndal, Sweden.; ^6^ Department of Neurodegenerative Disease, UCL Institute of Neurology, Queen Square, London, UK.; ^7^ UK Dementia Research Institute at UCL, London, UK.; ^8^ Hong Kong Center for Neurodegenerative Diseases, Clear Water Bay, Hong Kong, China.

## Abstract

Immunological control of viral infections in the brain exerts immediate protection and also long-term maintenance of brain integrity. Microglia are important for antiviral defense in the brain. Here, we report that herpes simplex virus type 1 (HSV1) infection of human induced pluripotent stem cell (hiPSC)–derived microglia down-regulates expression of genes in the TREM2 pathway. TREM2 was found to be important for virus-induced *IFNB* induction through the DNA-sensing cGAS-STING pathway in microglia and for phagocytosis of HSV1-infected neurons. Consequently, TREM2 depletion increased susceptibility to HSV1 infection in human microglia–neuron cocultures and in the mouse brain. TREM2 augmented STING signaling and activation of downstream targets TBK1 and IRF3. Thus, TREM2 is important for the antiviral immune response in microglia. Since *TREM2* loss-of-function mutations and HSV1 serological status are both linked to Alzheimer’s disease, this work poses the question whether genetic or virus-induced alterations of TREM2 activity predispose to post-infection neurological pathologies.

## Teaser

TREM2 is an important component of the antiviral immune response in microglia, which is actively targeted by HSV1.

## INTRODUCTION

Acute viral encephalitis is a severe disease, with often fatal outcome if untreated (*1*). Herpes simplex encephalitis (HSE) is the leading cause of viral encephalitis in the Western world and is caused by herpes simplex virus type 1 (HSV1) infection (*2*, *3*). HSV1 is a neurotropic human DNA virus that can reach the central nervous system (CNS) via retrograde axon transport after infecting peripheral sensory neurons. Although antiviral treatment is available for HSV1 and notably improves survival rates, post-HSE is often associated with serious sequelae. Between 50 and 80% of the adult population are HSV1-seropositive (*4*), and although HSE develops only in very few cases, epidemiological data have linked HSV1 infection with development of Alzheimer’s disease (AD) (*5*, *6*). Together, the available data therefore suggest that immunological control of HSV1 infection in the CNS is important for immediate protection of the brain and for long-term maintenance of brain integrity.

The early innate immune response is crucial to restrict HSV1 spread in the CNS. Microglia are the primary resident immune cells of the brain and protect the brain parenchyma against pathogen invasions through key macrophage functions, such as the release of antiviral type I interferons (IFN-I), cytokines, and phagocytosis of cell debris. Depletion of microglia increases viral spread in the CNS and consequently decreases the survival rates of mice infected with HSV1 (*7*–*9*). Optimal sensing and immune activation by microglia therefore require both continuous sampling of the local environment for danger signals and pathogen sensing by a broad repertoire of innate pattern recognition receptors (PRRs).

For the sensing, microglia express high levels of numerous PRRs, including the cytosolic DNA sensor cyclic guanosine monophosphate (GMP)–adenosine monophosphate (AMP) synthase (cGAS). cGAS detects and binds to HSV1 DNA, leading to 2′3′-cGAMP production and binding to stimulator of interferon genes (STING), which activates TANK-binding kinase 1 (TBK1) and interferon regulatory factor 3 (IRF3) (*10*, *11*). Activated IRF3 translocates to the nucleus to activate the transcription of IFN-I genes, including *IFNB*. Consequently, numerous IFN-stimulated genes (ISGs) are induced to exert direct antiviral activity through interference with specific steps in the viral replication cycle. Additionally, cGAS induces expression of inflammatory cytokines, such as interleukin-6 (IL-6) and tumor necrosis factor–α (TNF-α), via the nuclear factor κB (NF-κB) pathway. We previously reported that cGAS and STING are highly expressed in microglia, which use this pathway to produce the bulk of IFN-I in the HSV1-infected mouse brain (*12*). In humans, functional deficits in TBK1 or IRF3 are associated with HSE susceptibility (*13*, *14*), and several viral mechanisms to antagonize STING signaling have been identified (*15*–*18*). Collectively, this suggests that the cGAS-STING signaling axis plays an important role in control of HSV1 infection in the CNS.

For the sampling of the brain during both homeostatic and pathological conditions, microglia use a panel of receptors that bind a broad range of charged and hydrophobic molecules. These include scavenger receptor A-1, CD36, receptor for advanced glycation end products (RAGE), and triggering receptor expressed on myeloid cells-2 (TREM2). TREM2 is primarily expressed by microglia and interacts with a wide range of ligands, including bacterial products, apoptotic cells, β-amyloid (Aβ), anionic lipids, and apolipoprotein E (apoE) (*19*). Ligand engagement by TREM2 triggers downstream signaling through the adaptor protein DNAX activation protein 12 (DAP12) and recruitment and activation of the protein kinase SYK. In addition, TREM2 activity can be regulated via proteolytic processing of the receptor, which releases a soluble TREM2 fragment (sTREM2) (*20*), the function of which remains to be fully elucidated. In humans, loss-of-function mutations of *TREM2* or *DAP12* result in Nasu-Hakola disease, a serious disorder characterized by presenile dementia and bone cysts (*21*, *22*). Gene variants causing reduced function of TREM2 increase the risk to develop AD several fold (*23*, *24*). Less is known about the role of TREM2 during viral CNS infections. Studies investigating other neurotropic viruses, such as HHV-6A and HIV, suggest a link between altered TREM2 expression, inflammation, and neurocognitive disorders (*25*, *26*). However, the role of TREM2 during CNS infection with HSV1 is unexplored.

The aim of this study was to explore how HSV1 modulates gene expression in human microglia and to determine the functional impact on control of HSV1 infection. Here, we report that HSV1 infection of human induced pluripotent stem cell (hiPSC)–derived microglia selectively down-regulates expression of genes in the TREM2 pathway. TREM2 was found to be important for viral activation of cGAS-STING signaling in microglia and corresponding induction of the antiviral IFN-I response. Hence, TREM2 depletion in microglia and mice increased susceptibility to HSV1 infection both in human microglia–neuron cocultures and in vivo, respectively. These data identify TREM2 as a component of the antiviral immune response in microglia, which is actively targeted by HSV1.

## RESULTS

### HSV1 selectively blocks transcripts of the TREM2 pathway in hiPSC-derived microglia

**Fig. 1.**
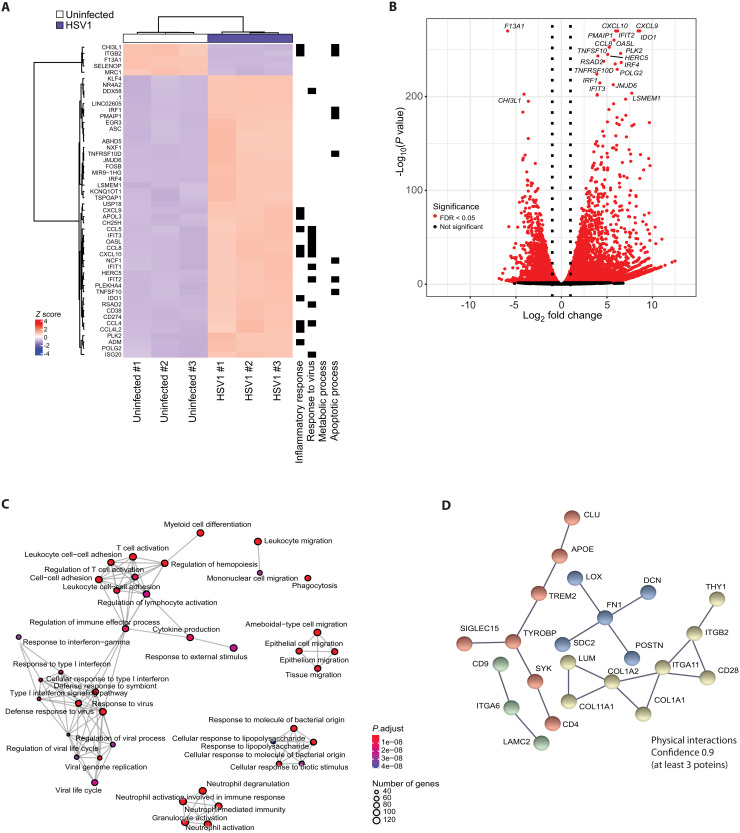
RNA-seq of HSV1-infected microglia. (**A** to **D**) hiPSC-derived microglia were infected with HSV1 (MOI 1) for 24 hours. (A) Hierarchical clustering of genes differentially expressed in HSV1-infected versus uninfected microglia. Top 50 genes are shown. Biological function annotated with the regulated genes shown to the right. (B) Volcano plot of differentially expressed genes in uninfected versus HSV1-infected microglia [red; false discovery rate (FDR) < 0.05]. The top 20 differentially regulated genes are indicated. (C) Gene ontology enrichment plot in the category biological processes of the top 50 differentially expressed genes. (D) Network representation of the protein-protein interactions of the 200 most down-regulated genes using STRING analysis. Nodes including at least 3 proteins (based on protein interaction data, confidence 0.9) are represented with different colors for each node.

We previously reported that microglia play a central role in early sensing of HSV1 infection in the brain, and activation of host defense in mice (*9*, *12*), and recently established that hiPSC-derived microglia are a powerful model system to study the antiviral immune response to herpesvirus infection (*18*, *27*). The differentiation workflow from iPSCs to microglia and cortical neurons is illustrated in fig. S1A. Using bulk RNA sequencing (RNA-seq) of hiPSC-derived microglia, we found that HSV1 infection profoundly altered microglial gene expression, with a large proportion of the genes being affected (fig. S1, B and C, and table S1). A broad panel of antiviral and proinflammatory genes was significantly up-regulated (Fig. 1, A and B). Gene ontology (GO) analysis suggests this response to affect various processes, most notably antiviral response, leukocyte cell-cell interactions, and T cell activation (Fig. 1C). However, we also noted that a subset of the genes annotated to “response to virus” was down-regulated (fig. S1C), and this included a panel of ISGs, which were either down-regulated or only modestly up-regulated (fig. S1D). To explore whether some of the down-regulated genes were functionally related, we performed STRING analysis of the 200 most down-regulated genes (table S2). The analysis suggested four interaction nodes in this group of transcripts (Fig. 1D). One of these included several components of the TREM2 pathway, including *TREM2*, *TYROBP* (*DAP12*), *APOE*, and *SYK*. Collectively, HSV1 strongly activates antiviral transcription programs in human microglia but also inhibits a subset of transcripts, including mRNAs encoding multiple components of the TREM2 pathway.

### HSV1 infection down-regulates microglial TREM2 expression in hiPSC-derived microglia

**Fig. 2.**
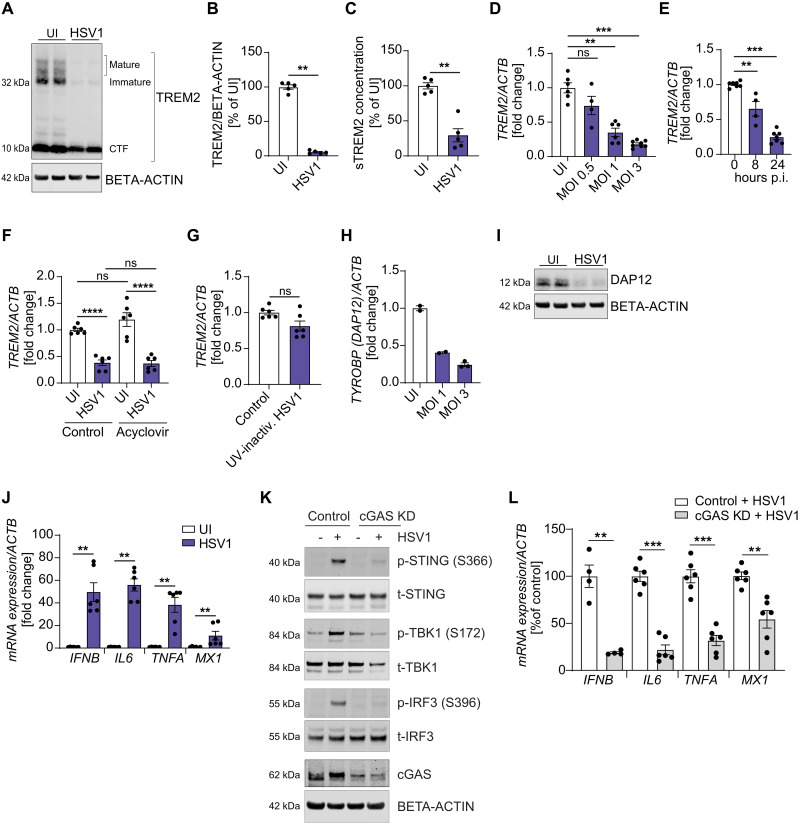
HSV1 down-regulates microglial TREM2 expression. (**A** to **C**) hiPSC-derived microglia were infected with HSV1 (MOI 1) for 24 hours. (A) Representative immunoblot of full-length TREM2 (TREM2) and C-terminal fragment (CTF). (B) Quantification of full-length TREM2 in uninfected (UI) microglia versus microglia infected with HSV1 (MOI 1) for 24 hours. (C) sTREM2 levels in cell supernatants of UI versus HSV1-infected microglia 24 hours after infection. (**D**) *TREM2* mRNA levels 24 hours after infection with HSV1 at different MOIs as indicated. (**E**) *TREM2* mRNA levels 8 and 24 hours after infection (p.i.) with HSV1 (MOI 1). (**F**) *TREM2* mRNA levels 24 hours after infection with HSV1 MOI 1 with and without 50 μM acyclovir. (**G**) *TREM2* mRNA 24 hours after incubation with and without UV-inactivated HSV1 (MOI 1). (**H**) *TYROBP* (*DAP12*) mRNA levels 24 hours after infection with HSV1 at different MOIs as indicated. (**I**) DAP12 protein levels 24 hours after infection with HSV1 (MOI 1). A representative immunoblot is shown. (**J**) *IFNB*, *IL6*, *TNFA*, and *MX1* mRNA levels in UI microglia versus HSV1-infected microglia 24 hours after infection (MOI 1). (**K** and **L**) cGAS was knocked down in hiPSC-derived microglia using siRNA. (K) Control and cGAS KD microglia were infected with HSV1 (MOI 3) for 5 hours. Representative immunoblots for the cGAS-STING signaling pathway and cGAS are shown. (L) *IFNB*, *IL6*, *TNFA*, and *MX1* mRNA levels were analyzed in control versus cGAS KD microglia 24 hours after infection with HSV1 (MOI 1). All figures represent at least two to three independent experiments; data are presented as mean ± SEM; *P* values were calculated by one-way ANOVA with Tukey’s multiple comparisons test (F) and Mann-Whitney *U* test (B to E, G, H, J, and L). **P < 0.001; ***P < 0.0005; ****P < 0.0001.

We confirmed that TREM2 expression is significantly reduced in HSV1-infected microglia (Fig. 2). The levels of cellular full-length immature and mature TREM2 protein as well as both of the proteolytic cleavage TREM2 products, i.e., the C-terminal fragment and secreted sTREM2, decreased substantially in cultures with HSV1-infected microglia (Fig. 2, A to C). *TREM2* mRNA decreased in a virus dose-dependent and time-dependent manner (Fig. 2, D and E). We observed this down-regulation with different HSV1 strains and in microglia differentiated from a separate iPSC line (fig. S2, A to C). In contrast to TREM2, and confirming the RNA sequencing data, several other central microglial genes were not affected (*MERTK*, *AXL*) or were up-regulated (*TMEM119*, *P2YR12*) during HSV1 infection (fig. S2, D and E and F and G). To characterize the viral requirements for down-regulation of TREM2 expression, we blocked viral replication using acyclovir treatment of cells or ultraviolet (UV) treatment of the virus. Acyclovir did not alter HSV1-induced *TREM2* down-regulation (Fig. 2F), whereas UV-inactivated HSV1 had lost the ability to alter *TREM2* expression levels (Fig. 2G). To test if HSV1 infection affects *TREM2* RNA stability, we used actinomycin D to block mRNA synthesis. *TREM2* mRNA decay occurred notably faster in the presence of HSV1 (fig. S2H). The virion host shutoff gene (UL41) of HSV1 encodes a virion component that induces degradation of specific host mRNAs. We observed only a slight rescue of *TREM2* expression when using the ΔUL41 HSV1 mutant compared with wild-type (WT) HSV1 (fig. S2I), suggesting that RNA degradation via this pathway is not the major mechanism exerting TREM2 down-regulation. Finally, we confirmed a substantial down-regulation of *TYROBP (DAP12)* mRNA and DAP12 protein (Fig. 2, H and I), thus validating the RNA sequencing data showing viral targeting of several components of the TREM2 pathway.

### HSV1 induces innate immune responses through the cGAS-STING pathway in human iPSC-derived microglia

As expected, HSV1 infection strongly induced *IFNB*, *IL6*, *TNFA*, and the ISG *MX1* in hiPSC-derived microglia (Fig. 2J). We have previously shown in mice that microglia sense HSV1 infection in the CNS through the cGAS-STING pathway to activate the antiviral program including type I IFNs (*9*, *12*). Here, we demonstrated that this is also the case in human microglia. Knockdown of *cGAS* substantially impaired HSV1 sensing as shown by decreased phosphorylation of STING, TBK1, and IRF3 in response to HSV1 infection (Fig. 2K and fig. S3D). Consequently, stimulation of *IFNB*, *IL6*, *TNFA*, and *MX1* was markedly impaired (Fig. 2L). Together, we observed robust down-regulation of TREM2 protein and mRNA in HSV1-infected human microglia and showed that the cGAS-STING pathway is essential for activating the antiviral program in human microglia.

### Depletion of microglial TREM2 expression impairs the innate immune response to HSV1 infection in hiPSC-derived microglia

**Fig. 3.**
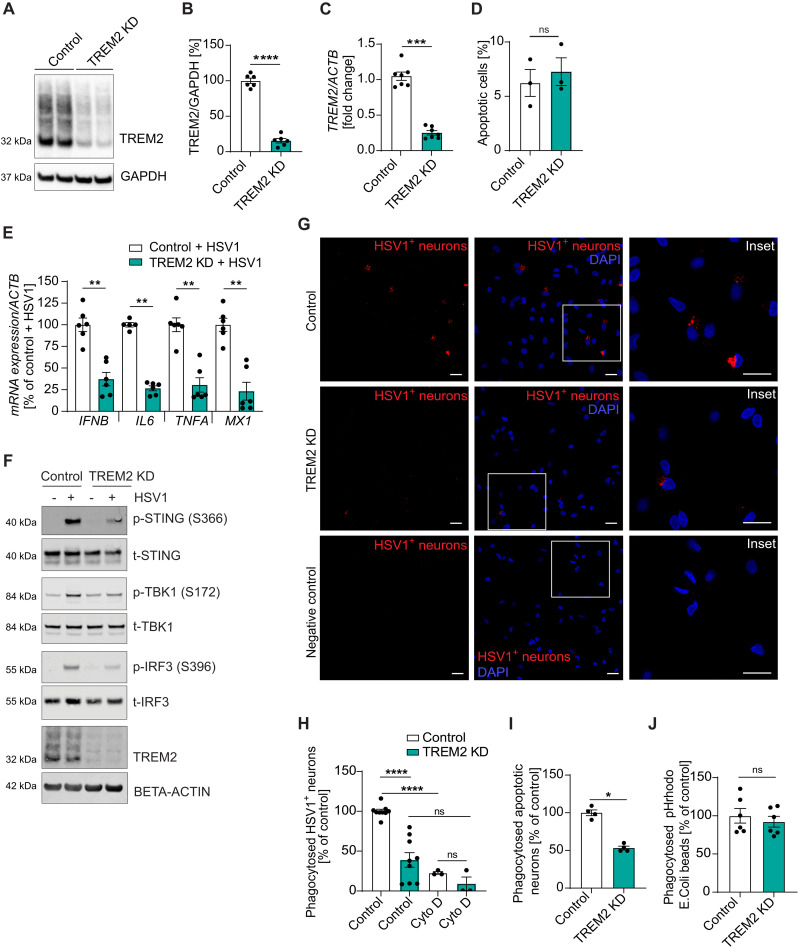
Depletion of TREM2 expression in microglia impairs innate immune responses to HSV1 in vitro. (**A** to **J**) TREM2 was knocked down in hiPSC-derived microglia using siRNA. Cells were used for experiments 4 days after transfection. (A) A representative immunoblot of full-length TREM2 (TREM2) of control and TREM2 KD microglia is shown. (B) Quantification of full-length TREM2 protein in control versus TREM2 KD microglia. (C) Quantification of *TREM2* mRNA in control and TREM2 KD microglia. (D) Apoptotic cells in control and TREM2 KD cultures were stained using an annexin V antibody and quantified by flow cytometry. (E) *IFNB*, *IL6*, *TNFA*, and *MX1* mRNA levels were analyzed in control versus TREM2 KD microglia 24 hours after infection with HSV1 (MOI 1). (F) Control and TREM2 KD microglia were infected with HSV1 (MOI 3) for 5 hours. Representative immunoblots are shown. (**G** and **H**) hiPSC-derived neurons were infected with HSV1 (MOI 1) for 24 hours, stained with a fluorescent cell tracker, and added to control and TREM2 KD microglia for 15 hours. (G) Cells were fixed and imaged using a confocal microscope (red, HSV1-infected neurons; blue, DAPI). The negative control were untreated cells. Scale bar, 20 μm. (H) Cells that had phagocytosed HSV1-infected neurons were quantified by flow cytometry. Cytochalasin D (Cyto D; 5 μM) was added to block phagocytosis. (**I**) Control and TREM2 KD microglia were incubated with fluorescent cell tracker–stained apoptotic neurons for 15 hours. Cells that had phagocytosed apoptotic neurons were quantified by flow cytometry. (**J**) Control and TREM2 KD microglia were incubated with pHrodo–*E. coli* beads for 3 hours. Phagocytosis of pHrodo–*E. coli* beads was quantified using a microplate reader. All figures represent two to three independent experiments; data are presented as mean ± SEM; *P* values were calculated by Mann-Whitney *U* test. *P < 0.05; **P < 0.001; ***P < 0.0005; ****P < 0.0001.

To determine the role of TREM2 during early HSV1 infection in hiPSC-derived microglia, we depleted *TREM2* using RNA interference (RNAi). The knockdown efficiency for full-length TREM2 was ~75% (Fig. 3, A to C, and fig. S3A), and sTREM2 was significantly decreased as well (fig. S3B). We did not observe a difference in cell viability in TREM2 knockdown (TREM2 KD) cells compared with controls (Fig. 3D). To determine whether the innate immune response to HSV1 infection is affected by TREM2 KD, we measured mRNA levels of *IFNB*, *IL6*, *TNFA*, and *MX1* (Fig. 3E). Strikingly, we observed a significantly decreased innate immune response in TREM2-depleted microglia. Similar results were found with microglia differentiated from a separate iPSC line (fig. S3C). Accordingly, we found impaired activation of the cGAS-STING pathway as shown by decreased phosphorylation of STING, TBK1, and IRF3 (Fig. 3F and fig. S3D). As sTREM2 has been suggested to exert biological functions such as protection against amyloid pathology (*28*), we then tested whether replenishing sTREM2 might be able to compensate for the loss of TREM2. Treatment with sTREM2 had no effect on *IFNB* or *MX1* but induced the proinflammatory cytokines *IL6* and *TNFA* (fig. S4, A to C), as shown previously (*29*). Likewise, in the presence of HSV1 infection, we observed only a small increase in *TNFA* (fig. S4D), suggesting that the decrease in sTREM2 during infection did not account for the effects observed.

Microglial protection against viral infection also involves phagocytosis of infected cells, especially neurons. To determine whether phagocytosis was impaired in TREM2-depleted microglia, we analyzed microglial phagocytosis of HSV1-infected neurons. Addition of fluorescently labeled HSV1-infected neurons to control microglia led to a prominent vesicular staining pattern (Fig. 3G, upper panel), whereas much less staining and fewer phagocytosing cells were observed in *TREM2* KD microglia (Fig. 3G, middle panel). No signal was observed in the negative control (Fig. 3G, lower panel). We further quantified the phagocytosis of HSV1-infected neurons using flow cytometry and found a significant decrease in the percentage of phagocytosed cells in TREM2 KD microglia (Fig. 3H). The phagocytosis inhibitor cytochalasin D almost completely blocked the phagocytosis of HSV1-infected neurons, indicating that phagocytosis is necessary for entry of infected neuron debris into microglia. As expected, decreased phagocytosis of apoptotic neurons was observed in TREM2 KD microglia (Fig. 3I), whereas the phagocytosis of *Escherichia coli* beads did not differ between control and TREM2 KD (Fig. 3J). As reported previously (*30*), this suggests a substrate-specific role of TREM2 in microglial phagocytosis and reveals that HSV1-infected neurons are phagocytosed in a TREM2-dependent manner. Together, these results show that the antiviral response in TREM2-depleted microglia is affected in several ways, including impaired IFNB induction via the cGAS-STING pathway, and clearance of infected neurons.

### TREM2 is essential for control of HSV1 infection in vitro and in vivo

**Fig. 4.**
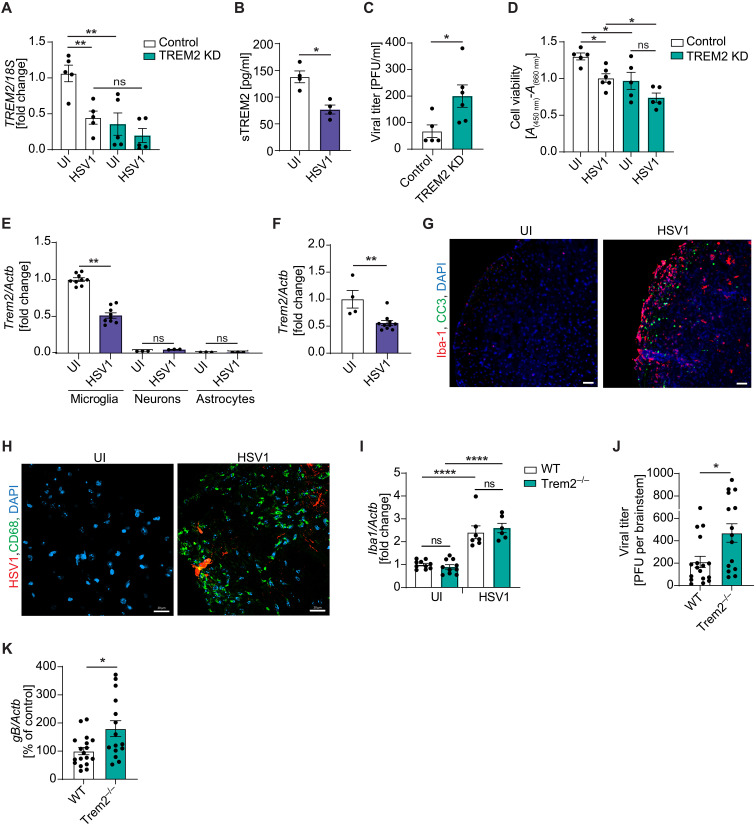
TREM2 is essential for control of HSV1 infection in vitro and in vivo. (**A**) *TREM2* mRNA levels were analyzed 24 hours after infection of hiPSC-derived cocultures of microglia and neurons with HSV1 (MOI 1). (**B**) sTREM2 levels were measured in supernatants of cocultures 24 hours after infection with HSV1 (MOI 1). (**C**) Viral titers in coculture supernatants 24 hours after infection with HSV1 (MOI 1) were quantified by viral plaque assay. (**D**) Cell viability was measured in cocultures 24 hours after infection with HSV1 (MOI 1) using the CyQUANT XTT assay. (**E**) *Trem2* mRNA levels were analyzed in primary murine microglia, neurons, and astrocytes 6 hours after infection with HSV1 (McKrae, MOI 3). (**F**) *Trem2* mRNA levels were analyzed in WT mouse brainstem 4 days after infection with HSV1 via the corneal route [2 × 10^6^ plaque-forming units (PFU)/cornea]. (**G** and **H**) WT mice were infected with HSV1 (2 × 10^6^ PFU/cornea), and brainstems were isolated and stained 5 days after infection. (G) Tissue sections were stained for Iba-1 (red) and CC3 (green). Scale bar, 50 μm. (H) Tissue sections were stained for HSV1 (red) and CD68 (green). Scale bar, 20 μm. (**I** and **K**) *Iba1* and *gB* mRNA levels were quantified in the brainstem of WT and Trem2^−/−^ mice 4 days after infection with HSV1 via the corneal route (2 × 10^6^ PFU/cornea). (**J**) Viral titers of isolated brainstems 4 days after infection with HSV1 via the corneal route (2 × 10^6^ PFU/cornea) were quantified by plaque assay. All figures represent two to three independent experiments; *n* = 15 to 18 per group of mice; data are presented as mean ± SEM; *P* values were calculated by one-way ANOVA with Tukey’s multiple comparisons test (A, D, and K) and Mann-Whitney *U* test (B, C, E, F, I, and J). *P < 0.05; **P < 0.001; ****P < 0.0001.

HSV1 is a neurotropic virus, and in the CNS, replication occurs predominantly in neurons. To investigate whether the impaired immune response in *TREM2* KD microglia affects the control of HSV1 infection in vitro, we cocultured hiPSC-derived cortical neurons and microglia (fig. S1A). As reported previously in mice (*12*), we observed that human microglia are potent producers of type I IFN (fig. S5A). As in microglia cultures, *TREM2* mRNA as well as secreted sTREM2 protein levels were significantly decreased in HSV1-infected cocultures (Fig. 4, A and B). Less viral replication and higher cell viability were observed in cocultures compared with neurons alone (fig. S5, B and C). Significantly higher viral replication occurred in TREM2-depleted cocultures, which was accompanied by decreased cell viability (Fig. 4, C and D).

To explore whether the phenomenon observed in human microglia was also observed in mice, we infected mouse brain cells in vitro or live animals through the corneal route, the latter leading to infection in the brainstem. We observed HSV1-induced *Trem2* down-regulation in primary mouse microglia and in the brainstem of HSV1-infected WT mice (Fig. 4, E and F). *Trem2* expression was not detected in astrocytes or neurons (Fig. 4E). In vivo, HSV1 infection significantly increased *Iba1* mRNA expression in the brainstem (Fig. 4I) and increased the number of cells positive for Iba-1 as well as cleaved caspase-3 (CC3), a marker for apoptosis (Fig. 4G). Furthermore, we found that HSV1 infection induced CD68, which is highly expressed by activated microglia (Fig. 4H). TREM2^+^ cells were abundantly present in the areas with HSV-1 infection, and some of these cells contained virus (fig. S5D). To determine whether TREM2 is essential for control of HSV1 infection in vivo, we compared viral load in WT versus *Trem2^−/−^* mice. Consistent with the data from the human coculture system, we observed significantly increased levels of infectious virus and viral mRNA in the brainstem of HSV1-infected *Trem2^−/−^* mice compared with WT (Fig. 4, J and K). We did not detect differences in *Iba1* expression in the brainstem between genotypes, suggesting that the observed infection phenotype was unlikely to be due to a difference in microglia frequency (Fig. 4I). Collectively, these findings suggest that TREM2 depletion leads to impaired control of HSV1 infection in vitro and in vivo.

### TREM2-depleted microglia respond normally to cGAMP

**Fig. 5.**
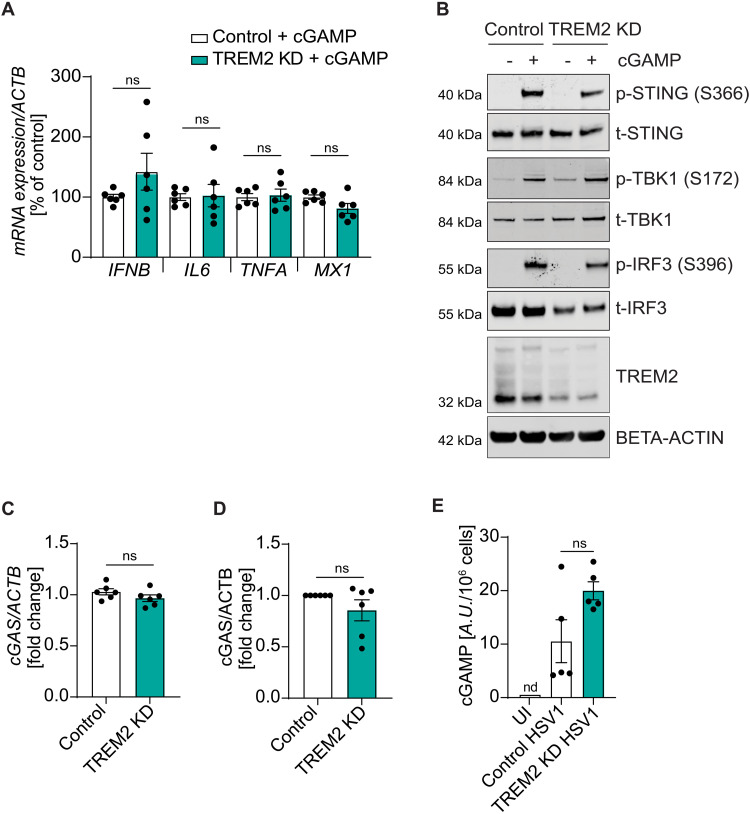
The antiviral program is not impaired in response to cGAMP in TREM2-depleted hiPSC-derived microglia. (**A** and **B**) Control and TREM2 KD microglia were stimulated with 100 μM cGAMP for 5 hours. (A) *IFNB*, *IL6*, *TNFA*, and *MX1* mRNA levels. (B) Representative immunoblots of the cGAS-STING axis and TREM2. (**C**) *cGAS* mRNA levels are shown in control and TREM2 KD microglia. (**D**) Quantification of cGAS protein levels in control and TREM2 KD microglia. (**E**) Quantification of cGAMP levels in HSV1-infected control versus TREM2 KD microglia 24 hours after infection. cGAMP was not detectable (nd) in UI cells; instead, the detection limit is shown. A.U., arbitrary units. All figures represent two to three independent experiments; data are presented as mean ± SEM; *P* values were calculated by Mann-Whitney *U* test.

As we observed impaired activation of the cGAS-STING pathway by HSV1 in TREM2-depleted microglia (Fig. 3F and fig. S3D), we wanted to test the functionality of this viral sensing pathway. To this end, we stimulated microglia with cyclic GMP-AMP (cGAMP), which directly activates STING. We found no significant difference in mRNA levels of *IFNB*, *IL6*, *TNFA*, and *MX1* in cGAMP-stimulated control versus TREM2-depleted microglia (Fig. 5A). Accordingly, we did not observe a difference in phosphorylation of STING, TBK1, and IRF3 in cGAMP-stimulated control versus TREM2-depleted microglia (Fig. 5B and fig. S6A). Also, we found no difference in *cGAS* mRNA or cGAS protein expression between control and TREM2 KD microglia (Fig. 5, C and D). In addition, we observed that HSV1-induced cGAMP accumulation in microglia was not affected by knockdown of TREM2 (Fig. 5E), whereas STING phosphorylation at S366 was reduced (Fig. 3F). In uninfected cells, cGAMP was below detection limit, which was more than 15 times lower than HSV1-induced cGAMP accumulation (Fig. 5E). These results suggest that cGAS-STING signaling is not intrinsically impaired in TREM2 KD microglia, but specifically in HSV1-infected cells, and this manifests in impaired activation of the pathway between cGAMP production and STING phosphorylation.

### Modulation of TREM2 activity via SYK modulates the innate immune response to HSV1 infection in microglia

**Fig. 6.**
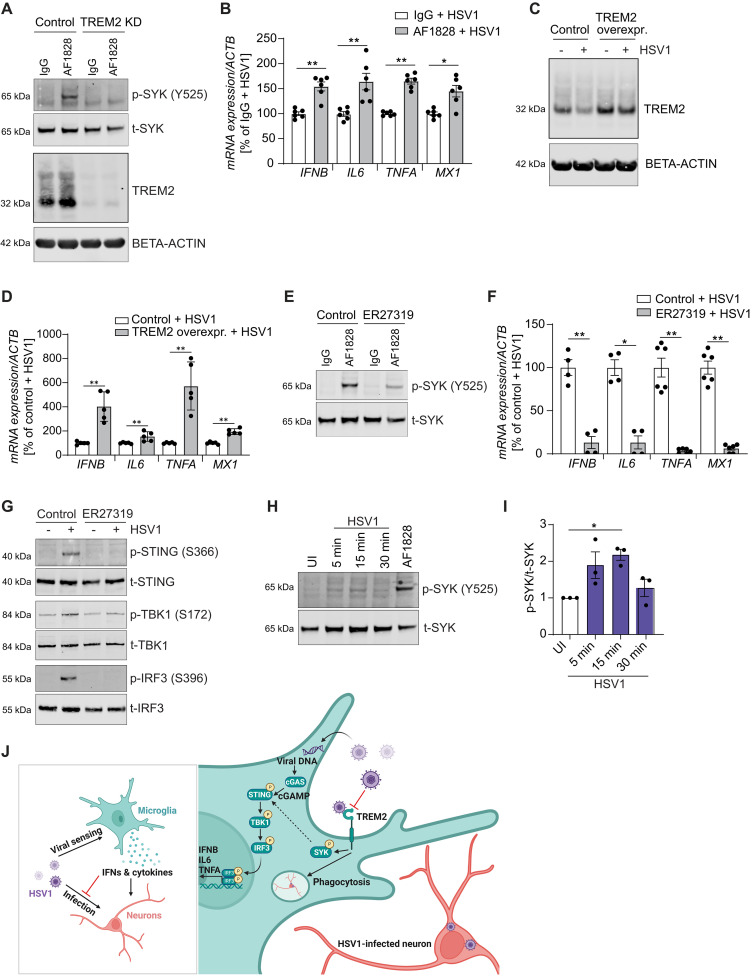
Modulation of TREM2 activity via SYK modulates the innate immune response to HSV1 infection in hiPSC-derived microglia. (**A**) Control and TREM2 KD microglia were incubated with control IgG or the TREM2-activating antibody AF1828 (0.8 μg/ml) for 15 min. Representative immunoblots of p-SYK and TREM2 are shown. (**B**) *IFNB*, *IL6*, *TNFA*, and *MX1* mRNA levels were analyzed in microglia after infection with HSV1 (MOI 1) in combination with control IgG or AF1828 for 8 hours. (**C**) Control and TREM2-overexpressing microglia were infected with HSV1 (MOI 1) for 24 hours. A representative immunoblot of TREM2 is shown. (**D**) *IFNB*, *IL6*, *TNFA*, and *MX1* mRNA levels were analyzed in control and TREM2-overexpressing microglia after infection with HSV1 (MOI 1) for 24 hours. (**E**) Microglia were incubated with control IgG or AF1828 (0.8 μg/ml) and with vehicle control or 5 μM ER27319 (SYK inhibitor) for 10 min. A representative immunoblot of p-SYK is shown. (**F**) Microglia were infected with HSV1 (MOI 1) with vehicle control or 5 μM ER27319 for 8 hours. *IFNB*, *IL6*, *TNFA*, and *MX1* mRNA levels are shown. (**G**) Microglia were incubated with vehicle control or 5 μM ER27319 and were infected with HSV1 (MOI 3) for 5 hours. Representative immunoblots for the cGAS-STING signaling pathway are shown. (**H** and **I**) Microglia were infected with HSV1 (MOI 5) for the indicated time points or stimulated with AF1828 (0.8 μg/ml) for 30 min. (H) Representative immunoblot. (I) Quantification of p-SYK/t-SYK from three independent experiments. (**J**) Illustration of the roles of microglial TREM2 during viral CNS infection. All figures represent two to three independent experiments; data are presented as mean ± SEM; *P* values were calculated by Mann-Whitney *U* test (B, D, and F) and one-way ANOVA with Tukey’s multiple comparisons test (I). *P < 0.05; **P < 0.001.

The protein kinase SYK, which is an immediate downstream target of TREM2, has been shown to be crucial for STING-mediated IFN induction by HSV1 (*31*). To determine whether TREM2 might affect the antiviral immune response via SYK, we modulated TREM2 signaling and SYK activity. First, we used a TREM2-activating antibody (AF1828), which induces downstream signaling via SYK activation. As microglia express Fc receptors, immunoglobulin G (IgG) was used as an isotype control. As previously reported (*32*), stimulation of human microglia with the AF1828 antibody rapidly induced SYK phosphorylation in control but not in TREM2 KD cells (Fig. 6A). Using this approach, we observed that stimulation with AF1828 amplified the early immune response to HSV1 infection, as shown by significantly increased levels of *IFNB*, *IL6*, *TNFA*, and *MX1* (Fig. 6B). Likewise, TREM2 overexpression boosted the early immune response to HSV1 infection, as shown by significantly increased levels of *IFNB*, *IL6*, *TNFA*, and *MX1* (Fig. 6, C and D). Next, we blocked TREM2 downstream signaling using a selective SYK inhibitor (ER27319) (Fig. 6, E and F). In the presence of ER27319, SYK phosphorylation was substantially decreased upon stimulation with AF1828 (Fig. 6E). Accordingly, we found significantly decreased levels of *IFNB*, *IL6*, *TNFA*, and *MX1* upon SYK inhibition in HSV1-infected microglia (Fig. 6F), as well as impaired activation of the cGAS-STING pathway, as shown by nearly absent phosphorylation of STING, TBK1, and IRF3 (Fig. 6G). In addition, we observed that HSV1 treatment itself induced a transient SYK phosphorylation in microglia (Fig. 6, H and I). Collectively, these results show that SYK activation downstream of TREM2 augments the antiviral response in microglia.

## DISCUSSION

Here, we report that HSV1 actively down-regulates the microglial receptor TREM2, and that this receptor plays an important role in full induction of the cGAS/STING-mediated IFN-I response to HSV1 infection. Depletion of TREM2 expression impaired the innate immune response to HSV1 infection, including type I IFN induction, and consequently promoted viral replication in human brain cell cultures and in mice. These data further support the idea of the essential role for microglia in early antiviral defense and provide mechanistic insight into the virus-cell interactions that govern the outcome of HSV1 infection in the brain.

Using bulk RNA sequencing, we found that HSV1 infection strongly induced antiviral and proinflammatory genes but also inhibited a subset of transcripts, including multiple components of the TREM2 pathway. In support of a role for TREM2 in the antiviral response, we found a marked decrease in the HSV1-induced activation of type I IFNs and ISGs in TREM2 KD microglia. Conversely, activation of TREM2 using an agonistic antibody or overexpression of TREM2 significantly elevated the antiviral immune response. Although we found that HSV1 infection robustly down-regulates microglial TREM2 expression, we observed substantial differences between control and TREM2 KD microglia in terms of antiviral defense mechanisms. These results indicate that TREM2 is essential for the immediate response to infection and that the virus seeks to target this response. This is in line with previous reports suggesting that the very early microglial immune response is crucial for control of HSV1 infection (*7*–*9*, *12*). We did not explore other down-regulated pathways, which were identified through RNA sequencing data analysis. However, it is interesting that two integrin-involving pathways were modulated by the virus, since integrins are important for the ability of microglia to migrate to sites of damage in the brain (*33*).

Herpesviruses encode a large number of genes involved in modulating cellular activities and thus facilitate establishment and maintenance of infection. Central for these activities is the inhibition of host gene transcription during productive infection. In the human microglia, we found that HSV1 infection led to decreased stability of *TREM2* RNA. To identify the mechanism would be of clinical relevance since it may reveal potential drug targets. Using acyclovir to block viral DNA replication and post-genome replication mRNA transcription, and UV inactivation of HSV1 to block all viral genome activity, we found that down-regulation of TREM2 requires active viral particles and occurs before viral DNA replication. Since productive HSV1 replication is rather limited in microglia, this may not be surprising. This was partially dependent on the HSV1 ribonuclease (RNase) viral host shutoff protein UL41, but most of the viral activity is not explained by the present data. In a recent phase II pilot trial, sTREM2 levels increased with valacyclovir administration in individuals with early-stage AD (*34*). In addition, TREM2 expression is also regulated by HIV, another virus with neurotropic properties (*25*, *26*). This suggests that TREM2 might be targeted by various viruses and the functional consequences require further investigations.

Our data demonstrate that it is not a “dead end” for HSV1 to enter microglia, although these cells cannot be productively infected. Rather, this interaction can impair the antiviral IFN-I response and hence promote the replication of virus that has entered more permissive cells, such as neurons.

As the primary immune cells of the brain, microglia recognize and remove injured and apoptotic cells. In our in vitro assay, phagocytosis of HSV1-infected neurons was found to be dependent on TREM2. Similar results have previously been reported for microglial phagocytosis of apoptotic neurons (*30*, *35*, *36*). HSV-infected neurons do not abundantly undergo apoptosis (*37*), and these data therefore illustrate how a range of different ligands can trigger TREM2-driven phagocytosis. In addition, the data from coculture and in vivo studies identified TREM2 as an important player in the control of HSV1 infection and spread in the brain. The observed effects of TREM2 loss of expression are likely a combination of impaired innate immune response, decreased clearance of infected neurons, and impaired recruitment of microglia to infected areas. It has been suggested that rapid recruitment of microglia is crucial to limit viral spread (*8*), and TREM2 deficiency has been demonstrated to impair chemotaxis and microglial responses to neuronal injury (*38*). Whether this also applies to HSV1-infected neurons requires further investigations.

We and others have previously shown in mice that the cGAS-STING pathway is essential for viral sensing and control of HSV1 infection in microglia (*9*, *12*, *39*). Here, we show that in hiPSC-derived microglia, *cGAS* depletion substantially impaired HSV1 sensing and immune activation. Strikingly, these same pathways were also impaired in TREM2 KD microglia. Using cGAMP as pathway agonist, we found that TREM2 is not intrinsically involved in STING signaling. Instead, TREM2 augments HSV1-induced cGAS-STING signaling downstream of cGAMP production and upstream of STING phosphorylation. Activation of SYK, which is initiated by TREM2-ligand interactions, has recently been shown to be required for STING-mediated IFN induction by HSV1 in different cell lines (*31*) and also to drive signal transducer and activator of transcription 1 (STAT1) activation in response to infection with RNA viruses (*40*). In hiPSC-derived human microglia, we observed that HSV1 infection led to rapid phosphorylation of SYK, and treatment with the SYK inhibitor ER27319 diminished the antiviral IFN immune response to HSV1 infection, which we found mainly to proceed through the cGAS-STING pathway. We therefore propose that TREM2 engagement on microglia by HSV1 or HSV1-infected cells triggers SYK activation, thus amplifying signaling through the cGAS-STING pathway by augmenting STING activity dependent on its phosphorylation on S366 (Fig. 6J). Upon viral entry into microglia, this response was blunted by down-regulation of *TREM2* mRNA levels, which consequently impairs the immediate antiviral host response. Recently, it has been demonstrated that hypofunctional TREM2 can be rescued by stimulating CLEC7A, a receptor that directly activates SYK, with an agonistic antibody (*41*). CLEC7A and potentially other receptors are potential therapeutic targets to boost microglial activation when TREM2 is impaired. This might be beneficial not only for HSV1 infection but also for other pathological brain conditions like AD.

HSV1 infection has been epidemiologically associated with development of AD, primarily in carriers of the *APOE* ɛ*4* allele (*42*). AD is the most common form of dementia and affects more than 10% of people over the age of 65 (*43*). Most of the genetic AD risk factors are implicated in microglial and innate immune cell functions (*44*), including a hypofunctional TREM2 allele, which increases the AD risk several fold (*23*, *24*). It is therefore tempting to speculate that the HSV1-induced down-regulation of TREM2 might promote or aggravate AD-related pathologies, especially amyloid plaque accumulation. Recent data highlight a role for TREM2 in activating and directing microglial responses in disease (*45*) and controlling the ability of microglia to surround, contain, or clear Aβ (*46*, *47*). Thus, microglial activation plays beneficial roles in prevention of AD, and impaired microglial activation results in an increased risk for AD. TREM2 has also been demonstrated to be crucial for microglia-mediated refinement of synapses in mice (*48*), thus highlighting another potential consequence of HSV1-mediated down-regulation of TREM2 expression. Given the growing interest in development of TREM2-activating antibodies as a potential therapeutic approach for AD (*49*, *50*), additional insights into TREM2 function add valuable information. Immune activation to infection is essential for eventual control of virus infection in the brain. However, the brain damage that occurs during HSE is caused by both viral replication and excessive inflammatory response (*51*). Our results not only suggest that elevating TREM2 function might be beneficial during acute HSV1 infection in the brain but also provide a possible mechanism for how viral reduction of TREM2 expression could affect Aβ pathology and accelerate disease processes.

The present work is mainly based on work in iPSC-derived human microglia and neurons, and in mice in vivo. Therefore, it does not demonstrate the relevance of the findings under natural infection in the human brain. This will require further studies, including exploration of the association of genetic variants of TREM2 with HSV1 infection in the brain. On the mechanistic level, it is a limitation of our work that the molecular mechanisms of viral destabilization of *TREM2* mRNA and of viral activation of TREM2-SYK signaling were not fully explained. DNA, different lipids, and complex carbohydrates, which are present on viral particles and stressed cells, have been reported as TREM2 agonists (*19*), and further studies are needed to uncover the HSV1-microglia interactions in more detail.

Together, we here demonstrate that the TREM2 pathway is down-regulated by HSV1 infection in microglia, and that this innate immune pathway plays a central part in the early antiviral immune response to HSV1 infection in the brain. Therefore, specific immunomodulatory strategies combined with antiviral therapy represent a promising path for better control of this devastating disease and for maintenance of brain integrity.

## MATERIALS AND METHODS

### Viruses and reagents

A neurovirulent clinical HSV strain was used for most in vitro experiments unless indicated otherwise: HSV1 2762 was isolated from the brain of a patient with fatal HSE during a clinical trial of acyclovir treatment (*52*). In addition, a KOS WT and a KOS mutant strain (ΔUL41) were used. For in vivo and primary murine cell in vitro experiments, the McKrae strain was used. The titers of the stocks used were determined by plaque assay on Vero cells [American Type Culture Collection (ATCC)]. AF1828 was from R&D Systems, control IgG was from Santa Cruz Biotechnology (sc-2028), 2′3′-cGAMP was from InvivoGen, human recombinant sTREM2 was from Sino Biological, and acyclovir was from Pfizer.

### Generation of human hiPSC-derived microglia

Two human iPSC lines, WTSIi015-A (EBiSC through Sigma-Aldrich) and ChiPSC22 (Takara Bio Europe), were used. WTSIi015-A iPSCs were used for most experiments unless indicated otherwise. iPSCs were maintained on Matrigel (Corning) in mTeSR1^+^ medium (STEMCELL Technologies). iPSC colonies were dissociated into single cells using TrypLE Express (Thermo Fisher Scientific). Per AggreWell 800 (STEMCELL Technologies) 24-well plate, 4 × 10^6^ iPSCs were seeded in 2 ml of embryonic body medium (EBM). EBM consisted of mTeSR1^+^ medium supplemented with 10 μM ROCK inhibitor, bone morphogenetic protein–4 (BMP-4) (50 ng/ml), stem cell factor (SCF) (20 ng/ml), and vascular endothelial growth factor (VEGF)–121 (50 ng/ml) (all from PeproTech). Cells were cultured for 4 days in Aggrewells to form embryonic bodies (EBs) with half-medium change (1 ml) every day. EBs were harvested using an inverted cell strainer (40 μm), and around 15 EBs were plated per six-well plate in hematopoietic medium (HM). HM consisted of X-VIVO 15 medium (Lonza) supplemented with 2 mM GlutaMAX, penicillin (100 U/ml), streptomycin (100 μg/ml), 55 μM β-mercaptoethanol, human macrophage colony-stimulating factor (M-CSF) (100 ng/ml) (PeproTech), and human IL-3 (25 ng/ml) (PeproTech). Every 7 days, 2 ml of medium was replaced by fresh HM. After around 30 days, primitive macrophage precursors could be harvested during the medium change and plated in microglia medium (MiM) at a density of 10^5^ cells/cm^2^. MiM consisted of Advanced DMEM F12 medium (Gibco) supplemented with 2 mM GlutaMAX, penicillin (100 U/ml), streptomycin (100 μg/ml), 55 μM β-mercaptoethanol, human IL-34 (100 ng/ml) (PeproTech), and human granulocyte-macrophage colony-stimulating factor (GM-CSF) (10 ng/ml) (PeproTech). Finally, cells were differentiated in MiM for 6 to 9 days with full medium change every other day.

### Generation of human hiPSC-derived cortical neurons

One day before neuronal induction, WTSIi015-A iPSCs were passaged using EDTA (Thermo Fisher Scientific) and pooled 2:1. The following day, medium was switched to neural maintenance medium (NMM). NMM consisted of Dulbecco’s modified Eagle’s medium (DMEM)/F12 and neurobasal medium (1:1) supplemented with 1× N2 supplement, 1× B27 supplement, 50 μM 2-mercaptoethanol, 0.5× nonessential amino acids, 100 μM l-glutamine (all from Life Technologies), penicillin/streptomycin (2500 U/ml) (GE Healthcare), insulin (10 μg/ml), and 0.5 mM sodium pyruvate (both from Sigma-Aldrich). NMM was further supplemented with mouse Noggin/CF chimera (500 ng/ml) (R&D Systems) and 10 μM SB431542 (STEMCELL Technologies). The cells were maintained in NMM for 10 to 12 days. The cells were then dissociated in colonies using Dispase II (10 mg/ml) (Thermo Fisher Scientific) and seeded on laminin-coated plates (1 to 2 μg/cm^2^; Sigma-Aldrich) in NMM supplemented with fibroblast growth factor 2 (FGF2) (20 ng/ml) (PeproTech). The cells were kept in FGF2-supplemented medium for 4 to 5 days and then further passaged with dispase two times before day 25. After 25 days, the colonies were passaged and expanded using StemPro Accutase (Thermo Fisher Scientific) until day 35. Cells can be frozen between day 25 and day 30. On day 35, the cells were passaged a last time onto plates coated with laminin (1 to 2 μg/cm^2^) at a density of 5 × 10^4^ cells/cm^2^ in NMM. The cells were then cultured for 2 weeks before they were used for experiments or the start of cocultures with hiPSC-derived microglia.

### Coculture of hiPSC-derived microglia and hiPSC-derived cortical neurons

Primitive macrophage precursors, harvested during medium change, were directly added to hiPSC-derived cortical neurons at a density of 2.5 × 10^4^ cells/cm^2^. After 1 hour, the medium was changed to coculture medium (CoM). CoM consisted of DMEM/F12 and neurobasal medium (1:1) supplemented with 1× N2 supplement, 2 mM GlutaMAX, penicillin (100 U/ml), streptomycin (100 μg/ml), 55 μM β-mercaptoethanol, human IL-34 (100 ng/ml), and human GM-CSF (10 ng/ml). Cells were cocultured for 2 weeks with full medium change every second day.

### TREM2 overexpression

The human iPSC line WTSIi015-A was used for TREM2 overexpression. Cells were grown in six-well plates and transduced 1 ml of mTESR^+^ with lentiviral particles [multiplicity of infection (MOI) 5] containing an expression plasmid for TREM2 (LPP-A3422-Lv105; GeneCopoeia). To increase transduction efficiency, polybrene (5 μg/ml) was added. After 6 hours, an additional 1 ml of mTESR^+^ was added. Two days after transduction, selection was started using puromycin (0.5 μg/ml). Cells were subsequently passaged, and single-cell clones were analyzed for transgene expression. A few clones were selected and used for generation of human hiPSC-derived microglia. To compensate for potential clonal effects, data from two separate clones were pooled.

### siRNA-mediated knockdown

Human hiPSC-derived microglia were transfected with Lipofectamine RNAiMAX reagent (Life Technologies) according to the manufacturer’s protocol. A final small interfering RNA (siRNA) concentration of 200 nM was used. Cells were used for experiments on day 4 after transfection. The following Silencer siRNAs (Ambion) were used: control (siRNA ID: 4390843), TREM2 (siRNA ID: 289814), and cGAS (siRNA ID: 129127).

### Mice

C57BL/6 (WT) and *Trem2^−/−^* mice were bred at the University of Aarhus (Denmark). *Trem2^−/−^* mice were purchased from The Jackson Laboratory. Isoflurane (Abbott) or a mixture of ketamine (MSD Animal Health) and xylazine (Rompun Vet) was used to anesthetize mice. All described animal experiments have been reviewed and approved by the Danish government authorities and hence comply with Danish laws (ethics approval Nr. 2016-15-0201-01085). All efforts were made to minimize suffering, and mice were monitored daily during infection. The mice were not randomized, but after HSV1 infection, information about mouse strain and treatment was blinded to the investigators. No animals were excluded from the analysis. Chow and water were provided ad libitum.

### Murine ocular and HSV1 infection model

Age-matched, 6- to 7-week-old male mice were anesthetized with intraperitoneal injection of a mixture of ketamine (100 mg/kg body weight) and xylazine (10 mg/kg body weight). We tested male and female mice and did not find any sex differences in any of the readouts used in the current study. Both corneas were scarified in a 10 × 10 crosshatch pattern with a 25-gauge needle, and mice were inoculated with HSV1 (strain McKrae, the dosage used is indicated in the figure legends) in 5 μl of infection medium [DMEM containing penicillin (200 IU/ml) and streptomycin (200 mg/ml)] or mock-infected with 5 μl of infection medium.

### Plaque-forming unit assay

Vero cells (ATCC) were used for plaque assays and maintained in MEM containing 5% fetal bovine serum (FBS), 1% penicillin, and 1% streptomycin. Vero cells were incubated with serial dilutions of cell supernatants. After 1 hour, 0.75% methylcellulose (Sigma) was added, and plates were incubated at 37°C. After 3 days, the plaques were stained and counted under the microscope.

Mouse brainstems were isolated and immediately put on dry ice. Brainstems were then homogenized in DMEM and pelleted by centrifugation at 1600*g* for 30 min. Supernatants were used for plaque assay as described (*12*).

### sTREM2 enzyme-linked immunosorbent assay

sTREM2 was measured using an in-house electrochemiluminescent assay on the MESO QuickPlex SQ 120 instrument (MesoScale Discovery, USA) using a method developed by Kleinberger *et al.* (*53*) with modifications by Alosco *et al.* (*54*). Briefly, the capture antibody was biotinylated polyclonal goat anti-human TREM2 (0.25 μg/ml, R&D Systems), and the detector antibody was monoclonal mouse anti-human TREM2 (1 μg/ml, Santa Cruz Biotechnology). A standard curve for calculations of unknowns was constructed using recombinant human TREM2 (4000 to 62.5 pg/ml, Sino Biological), and samples were diluted 1:4 before being assayed.

### RNA isolation, RT-PCR, and qPCR

Tissues were homogenized with steel beads (Qiagen) in Tissuelyser (II) (Qiagen) in phosphate-buffered saline (PBS) and immediately used for RNA isolation. RNA from mouse brainstem or primary cell cultures was isolated using the High Pure RNA Isolation Kit (Roche). hiPSC-derived cells were lysed in RLT Plus buffer (Qiagen) supplemented with 4 mM dithiothreitol (DTT) (Sigma), and RNA was isolated using the RNeasy Mini Kit (Qiagen). cDNA was synthesized using the High-Capacity cDNA Kit (Thermo Fisher Scientific).

Quantitative polymerase chain reaction (qPCR) was performed using the following TaqMan Gene Expression Assays (Applied Biosystems): *Actb* (Mm00607939_s1), *Trem2* (Mm04209424_g1), *Iba1* (Mm Mm00479862_g1), *ACTB* (Hs01060665_g1), *18S* (Hs03003631_g1), *TREM2* (Hs00219132_m1), *IFNB* (Hs01077958_s1), *IL6* (Hs00174131_m1), *TNFA* (Hs00174128_m1), *MX1* (Hs00895598_m1), and *cGAS* (Hs00403553_m1). For human *HSV1 gB*, the following custom TaqMan primers and probe were used: forward primer 5′-GCAGTTTACGTACAACCACATACAGC-3′, reverse primer 5′-AGCTTGCGGGCCTCGTT-3′, and probe 56-FAM/CGGCCCAACATATCGTTGACATGGC/3BHQ_1 (IDT). For mouse *HSV1 gB*, the following custom TaqMan primers and probe were used: forward primer (5′-CGCATCAAGACCACTCCTC-3′), reverse primer (5′-AGCTTGCGGGCCTCGTT-3′), and probe (5′-CGGCCCAACATATCGTTGACATGGC-3′). mRNA levels of interest were normalized to the housekeeping gene *Actb/ACTB* or *18S* (as indicated) using the ΔΔ*C*_t_ method. *18S* was used as a reference gene for cocultures, as *ACTB* expression was consistently affected by HSV1 infection.

### RNA sequencing and data analysis

hiPSC-derived microglia were grown on 24-well plates at a density of 2 × 10^5^ cells per well and infected with HSV1 (MOI 1) for 24 hours. Cells were then lysed in RLT Plus buffer supplemented with 4 mM DTT. RNA was isolated using the RNeasy Micro Kit (Qiagen) including the DNA digestion step, which yielded ~1.5 μg of RNA. RNA-seq including bioinformatics analysis was performed at Omiics ApS (Denmark). Samples were ribosomal RNA (rRNA)–depleted and prepared for sequencing using SMARTer Stranded Total RNA Sample Prep Kit - HI Mammalian (Takara). Briefly, this kit first removes rRNA using RiboGone technology that specifically depletes nuclear rRNA sequences (5*S*, 5.8*S*, 18*S*, and 28*S*) and mitochondrial rRNA 12*S*. RiboGone oligos are hybridized to rRNA, which is cleaved using RNase H–mediated cleavage. First-strand synthesis is performed using random priming, adding an anchor for use with later PCR step. Template switching is used during the reverse transcription (RT) step and adds additional nontemplated nucleotides to the 3′ end of the newly formed cDNA. PCR is performed leveraging the nontemplated nucleotides and the added anchor sequence to produce Illumina compatible libraries. Prepared libraries were quality-controlled using the Bioanalyzer 2100 (Agilent) and qPCR-based concentration measurements. Libraries were equimolarly pooled and sequenced including 150–base pair (bp) paired end reads on an Illumina HiSeq sequencer. Sequencing data were preprocessed by removing adapter sequence and trimming away low-quality bases with a Phred1 score below 20 using Trim Galore (v0.4.1). Quality control was performed using FastQC, Picard, and MultiQC to ensure high-quality data. HSV1 genome (accession code JQ673480.1) was downloaded from National Center for Biotechnology Information (NCBI). The filtered RNA-seq data were mapped against the viral genome using STAR. Reads not mapping to the viral genome were extracted and mapped against the human genome (hg38/GRCh38) using STAR, and gene expression was quantified using featureCounts with gene annotations from Gencode release 38. Differential expression analysis was performed using DESeq2 package in R for human gene expression profiles.

GO analysis was done using the clusterProfiler R package (*55*). For GO analysis, all significantly differentially expressed genes [false discovery rate (FDR) < 0.05] were used except in cases were more than 3000 significant genes were found, in which case the 3000 most significant genes were used. Plotting was done in R.

### STRING analysis

To identify nodes in the virus-regulated transcripts, we used the protein-protein interaction analysis tool STRING (https://string-db.org/). The proteins corresponding to the 200 most down-regulated genes in the RNA-seq dataset were subjected to STRING analysis. The analysis included only physical subnetworks, and a confidence of at least 0.9 was used as cutoff criteria. At least three proteins were required to qualify for a node.

### Protein isolation and immunoblotting

hiPSC-derived microglia were grown on six-well plates for protein isolation. For signaling experiments, cells were infected with HSV1 (MOI 3) for 5 hours (unless indicated otherwise). For the analysis of HSV1-regulated protein expression, cells were infected with HSV1 (MOI 1) for 24 hours. Cells were washed twice with PBS and lysed on ice for 5 min in radioimmunoprecipitation assay (RIPA) buffer [20 mM tris-HCl (pH 7.5), 150 mM NaCl, 1 mM EDTA, 1% Triton X-100, 0.5% sodium deoxycholate, 0.1% SDS] supplemented with protease and phosphatase inhibitor cocktails (Roche). Samples were sonicated on ice for 10 min and centrifuged at 14,000*g* at 4°C for 10 min. Supernatants were collected and stored at −80°C for further use. For immunoblotting, samples were boiled at 95°C for 5 min under reducing conditions.

The following primary antibodies were used: TREM2 C-terminal (CS91068, Cell Signaling Technology), glyceraldehyde-3-phosphate dehydrogenase (GAPDH) (CS5174), p-STING S366 (CS50907, Cell Signaling Technology), t-STING (CS13647, Cell Signaling Technology), p-TBK1 S172 (CS5483, Cell Signaling Technology), t-TBK1 (CS3504, Cell Signaling Technology), p-IRF3 S396 (CS29047, Cell Signaling Technology), t-IRF3 (CS4302, Cell Signaling Technology), cGAS (CS79978), β-actin (CS3700, Cell Signaling Technology), p-SYK Y525/526 (CS2710, Cell Signaling Technology), and t-SYK (CS13198, Cell Signaling Technology). The following secondary antibodies were used: IRDye 800CW donkey anti-rabbit IgG and IRDye 680RD donkey anti-mouse IgG (both from LI-COR Biotechnology).

### Immunocytochemistry

Cells grown on Ibidi μ-slides (Ibidi) were washed twice in PBS and fixed in 4% paraformaldehyde for 20 min at room temperature, covered with PBS, and stored at 4°C until analysis. The cells were permeabilized with 0.3% Triton X-100 in tris-buffered saline (TBS) for 15 min at room temperature and incubated with blocking buffer (0.3% Triton X-100 and 5% donkey serum in TBS) for 1 hour at room temperature. Primary antibodies were diluted in blocking buffer. The following primary antibodies were used for staining: IBA1 (ab5076, Abcam), TUJ1 (ab14545, Abcam), and TREM2 (AF1828, R&D Systems). Samples were incubated with primary antibodies overnight at 4°C. Cells were washed three times in TBS and incubated for 1 hour with secondary antibodies diluted 1:500 in blocking buffer. Cells were washed three times in TBS, counterstained using DAPI (4′,6-diamidino-2-phenylindole), and mounted using Ibidi mounting medium (Ibidi). Samples were imaged using a Nikon A1 inverted confocal microscope.

### Immunohistochemistry

Immunohistochemistry was done as previously described (*37*). Briefly, mice were perfused, dissected brains were fixed in 4% formaldehyde and embedded in paraffin, and sections (7 μm) were cut. Sections were deparaffinized and rehydrolyzed, and antigens were retrieved for 30 min at 80°C using the Target Retrieval Solution (Dako). Sections were blocked with blocking buffer (1% bovine serum albumin and 0.3% Triton X-100 in TBS) for 1 hour at room temperature. Samples were incubated for 48 hours at 4°C with the following primary antibodies: HSV-1 (1:500; B0116, Dako, Cytomation), CC3 (1:300; CS9661, Cell Signaling Technology), Iba-1 (1:400; ab5076, Abcam), and Trem2 (1:50; AF1729, R&D Systems). As a negative control, we used secondary antibodies alone or an isotype control if the primary antibody was monoclonal. After several washes in TBS-TX (0.3% Triton X-100 in TBS), the sections were incubated with appropriate secondary antibodies coupled to Alexa Fluor 647, 568, or 488 (1:500; Invitrogen) for 1 hour at room temperature. Nuclei were stained with DAPI for 6 min. Finally, the sections were washed five times with TBS-TX and mounted with ProLong Gold. Sections were imaged on a Zeiss LSM 710, LSM 800 laser scanning microscope, and Leica Leitz DMRB fluorescence microscope. Zen 2012 acquisition software and ImageJ (National Institutes of Health) were used for imaging and analysis.

### cGAMP quantification

hiPSC-derived microglia were grown on six-well plates and infected with HSV1 (MOI 1) for 24 hours. Cells were washed twice with PBS, carefully scraped, and collected in 500 μl of PBS per well, and two wells were merged. Cells were counted and then centrifuged at 200*g* for 5 min. The supernatant was discarded, and 250 μl of internal standard solution [10 nM cGMP in acetonitrile:methanol (1:1)] was added to the cell pellet. Samples were vortexed and stored at −80°C until further analysis. Samples were extracted by sonication for 10 min followed by vortexing (1400 rpm) for an additional 10 min. Samples were centrifuged at 16,000*g* for 10 min, and supernatants were evaporated under a stream of nitrogen before reconstitution in 100 μl of acetonitrile:methanol (3:1). Quantification was performed using ultraperformance liquid chromatography coupled to tandem mass spectrometry (UPLC-MS/MS). Five microliters was injected onto a Waters zHILIC column (2.1 × 100; 1.7 μm particles) and separated using a gradient of acetonitrile with 0.1% formic acid as B-phase and water with 20 mM ammonium formate as A-phase. Detection was performed on a Sciex 7500 triple quadrupole mass spectrometer (Sciex). Quantification was made against an external calibration curve with a limit of detection of 0.1 nM.

### Cell viability assay

The CyQUANT XTT assay (Invitrogen) was used to measure cell viability. hiPSC-derived cocultures were infected with HSV1 as indicated. After 24 hours, 150 μl of medium was removed and stored at −80°C for further analyses. Sixty-five microliters of CyQUANT Mix was added to the remaining 100 μl, and cells were incubated for 3 hours. Cell supernatants were transferred to a 96-well plate, and absorbance (*A*) was measured at 450 and 660 nm. Specific absorbance was calculated by using the following formula: *A*_(450 nm)_ − *A*_(660 nm)_.

### Annexin V staining and FACS analysis

Annexin V–Alexa Fluor 488 conjugates (Thermo Fisher Scientific) were used for apoptosis detection according to the manufacturer’s instructions. Briefly, hiPSC-derived microglia were trypsinized, washed once with PBS at 400*g* for 5 min, and resuspended in annexin-binding buffer at 1 × 10^6^ cells/ml. Five microliters of annexin V conjugate was added per 100 μl of cell solution and incubated for 15 min at room temperature, and 400 μl of annexin-binding buffer was added. Cells were analyzed using FACSAria Fusion, the annexin V conjugate signal was detected in the fluorescein isothiocyanate (FITC) channel, and positive cells were quantified using the BD FACSDiva software.

### Phagocytosis assays

For analysis of phagocytosis of HSV1-infected neurons, hiPSC-derived neurons were infected with HSV1 (10 MOI). After 24 hours, cells were washed once with PBS and fresh NMM was added. Cells were subjected to UV irradiation for 10 min to inactivate HSV1. Cells were then trypsinized, centrifuged at 400*g* for 5 min, resuspended in PBS containing fluorescent cell tracker (2 μg/ml) (CellTracker CM-DiI Dye, Thermo Fisher Scientific), and incubated for 5 min at 37°C. Cells were washed twice with PBS at 400*g* for 5 min. The UV inactivation of the virus was confirmed by plaque assay. Cells were resuspended in MiM and added to the cultured hiPSC-derived microglia, at a 1:1 cell ratio, for 15 hours. For immunocytochemistry (ICC), microglia were washed twice with PBS and fixed in 4% paraformaldehyde for 20 min at room temperature followed by DAPI counterstaining and mounting. Samples were imaged using a Nikon A1 inverted confocal microscope. For fluorescence-activated cell sorting (FACS) analysis, microglia were trypsinized, washed twice with PBS at 400*g* for 5 min, and resuspended in 200 μl of PBS for FACS analysis using FACSAria Fusion. The cell tracker signal was detected in the phycoerythrin (PE) channel, and positive cells were quantified using the BD FACSDiva software.

For analysis of phagocytosis of apoptotic neurons, apoptosis was induced in hiPSC-derived neurons by adding 30 nM okadaic acid to the cell medium for 3 hours. Neurons were trypsinized, washed once in PBS, resuspended in PBS containing fluorescent cell tracker (2 μg/ml), and incubated for 5 min at 37°C. Cells were washed twice with PBS at 400*g* for 5 min, resuspended in MiM, and added to the cultured hiPSC-derived microglia at a 1:10 cell ratio for 3 hours. Microglia were prepared for FACS analysis as above.

For analysis of phagocytosis of pHrodo–*E. coli* beads (Thermo Fisher Scientific), beads were resuspended in live cell imaging solution (2 ml/vial of beads, Thermo Fisher Scientific) and briefly sonicated. hiPSC-derived microglia were grown in black clear bottom 96-well plates and incubated with 100 μl of bead solution for 3 hours. Phagocytosis of pHrodo–*E. coli* beads was quantified using a microplate reader and excitation/emission wavelengths of 560/585 nm. To inhibit phagocytosis, cells were incubated with 5 μM cytochalasin D (Thermo Fisher Scientific) 1 hour before experiments and during experiments.

### mRNA stability assay

Actinomycin D, which inhibits the synthesis of new mRNA, was used for the assessment of mRNA decay. hiPSC-derived microglia were incubated with actinomycin D (5 μg/ml) for the indicated time points in the presence and absence of HSV1 (MOI 3). RNA was isolated, followed by cDNA synthesis and qPCR. *TREM2* mRNA expression was quantified relative to time point 0.

### Statistical analyses

For statistical analysis of data, we used two-tailed Student’s *t* test when the data exhibited normal distribution, and Mann-Whitney *U* test when the dataset did not pass the normal distribution test or normal distribution could not be tested. When comparing more than two groups, multiple-comparison one-way analysis of variance (ANOVA) was used with Tukey’s multiple-comparison test. All experimental data were reliably reproduced in two or more independent experiments. Individual data points represent biological replicates. GraphPad Prism 8 software and R were used for statistical analyses. No measurement was excluded for statistical analysis.
