# Supplementary material for: TREM2 is down-regulated by HSV1 in microglia and involved in antiviral defense in the brain
Source: Sci Adv. 2023 Aug 18;9(33):eadf5808. doi: 10.1126/sciadv.adf5808 (PMC10438464; doi:10.1126/sciadv.adf5808)
Supplement: Supplementary file 1 — Figs. S1 to S6 Legends for data S1 and S2 [file sciadv.adf5808_sm.pdf]

Science Advances
AAAS

Supplementary Materials for

TREM2 is down-regulated by HSV1 in microglia and involved in antiviral defense in the brain

Stefanie Fruhwürth *et al.*

Corresponding author: Søren R. Paludan, srp@biomed.au.dk; Stefanie Fruhwürth, stefanie.fruhwurth@gu.se

Sci. Adv. **9**, eadf5808 (2023)
DOI: 10.1126/sciadv.adf5808

The PDF file includes:

Figs. S1 to S6
Legends for data S1 and S2

Other Supplementary Material for this manuscript includes the following:

Data S1 and S2

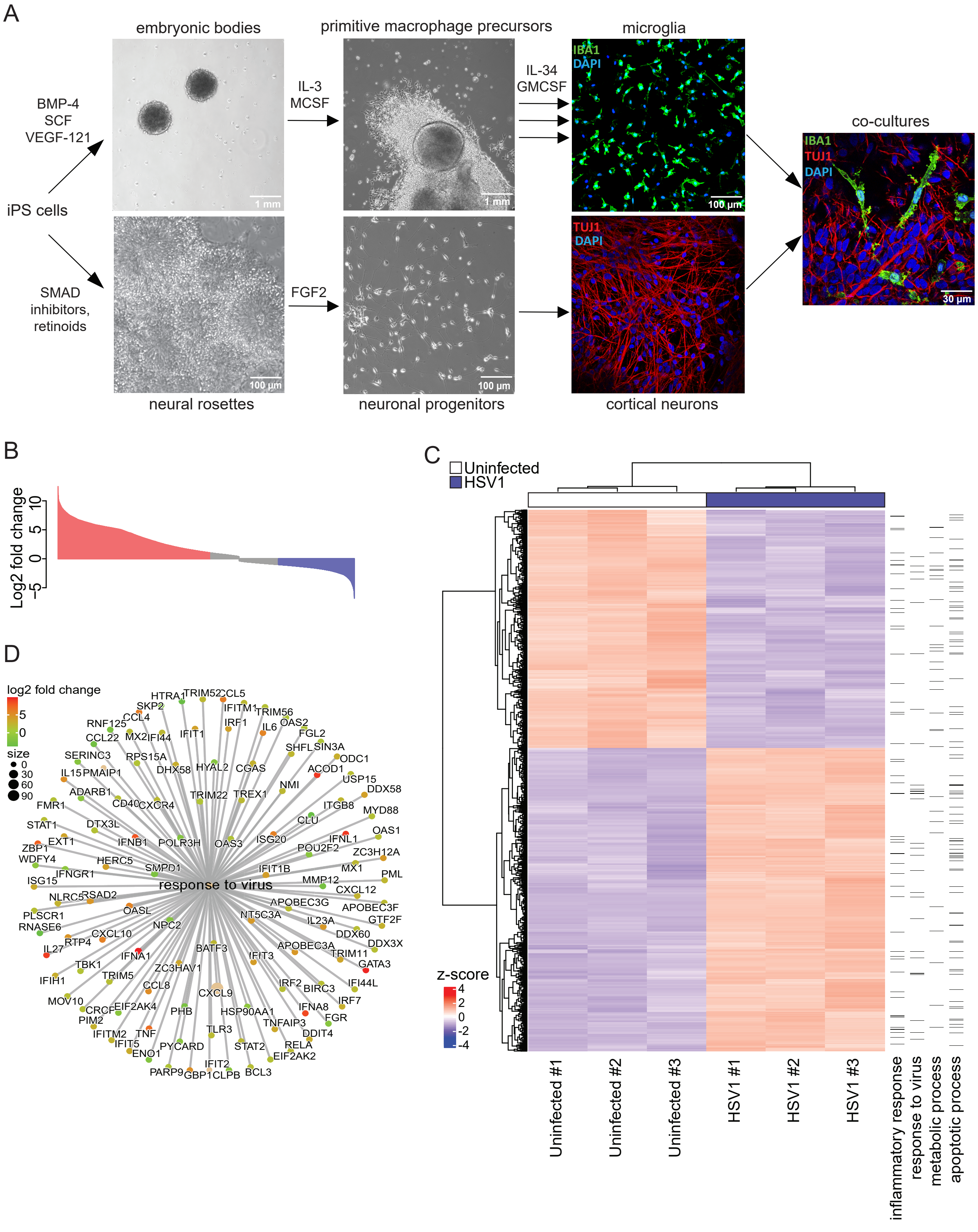
A
embryonic bodies
primitive macrophage precursors
microglia
BMP-4
SCF
VEGF-121
IL-3
MCSF
IL-34
GMCSF
IBA1
DAPI
1 mm
1 mm
100 μm
co-cultures
IBA1
TUJ1
DAPI
30 μm
iPS cells
SMAD
inhibitors,
retinoids
FGF2
TUJ1
DAPI
100 μm
100 μm
neural rosettes
neuronal progenitors
cortical neurons
B
Log2 fold change
10
5
0
-5
C
Uninfected
HSV1
z-score
4
2
0
-2
-4
Uninfected #1
Uninfected #2
Uninfected #3
HSV1 #1
HSV1 #2
HSV1 #3
inflammatory response
response to virus
metabolic process
apoptotic process
D
log2 fold change
5
0
size
0
30
60
90
response to virus
HTRA1 TRIM52 CCL5
SKP2 IFITM1 TRIM56
RNF125 CCL4 IRF1 IL6 OAS2
CCL22 MX2 IFI44 IFIT1 FGL2
SERINC3 SHFL SIN3A
RPS15A ODC1
IL15 PMAIP1 DHX58 HYAL2 CGAS ACOD1 USP15
ADARB1 NMI DDX58
FMR1 CD40 CXCR4 TRIM22 TREX1 ITGB8 MYD88
STAT1 DTX3L CLU OAS1
ZBP1 EXT1 IFNB1 POLR3H OAS3 ISG20 IFNL1
WDFY4 HERC5 POU2F2 ZC3H12A
IFNGR1 SMPD1 IFIT1B MX1 PML
ISG15 MMP12 CXCL12
NLRC5 RSAD2 APOBEC3G APOBEC3F
PLSCR1 OASL NT5C3A IL23A GTF2F
RNASE6 NPC2 DDX60
CXCL10 DDX3X
IL27 RTP4 BATF3 APOBEC3A
IFNA1 IFIT3 TRIM11
TBK1 ZC3HAV1 GATA3
IFIH1 TRIM5 IRF2 BIRC3 IFI44L
MOV10 CCL8 CXCL9 IRF7
CRCF EIF2AK4 PHB HSP90AA1 IFNA8
PIM2 TNFAIP3 FGR
IFITM2 TNF TLR3 DDIT4
IFIT5 PYCARD STAT2 RELA
ENO1 EIF2AK2
IFIT2
PARP9 GBP1 CLPB BCL3

Fig. S1. Stem cell differentiation workflow and RNA-seq data analysis of hiPSC-derived microglia. (**A**) Illustration of the differentiation workflow from hiPSCs towards microglia and cortical neurons is shown. Cells were stained for markers for microglia (IBA1, green), cortical neurons (TUJ1, red), and nuclei (DAPI, blue). Multiple arrows indicate continuous harvest of microglia. (**B**) Waterfall plot for RNA-seq of HSV1-infected microglia relative to uninfected microglia. (**C**) Hierarchical clustering of genes differentially expressed in uninfected versus HSV1-infected microglia. Biological function annotated with the regulated genes is shown on the right. (**D**) Differentially regulated genes in the gene ontology category “response to virus” in HSV1-infected microglia.

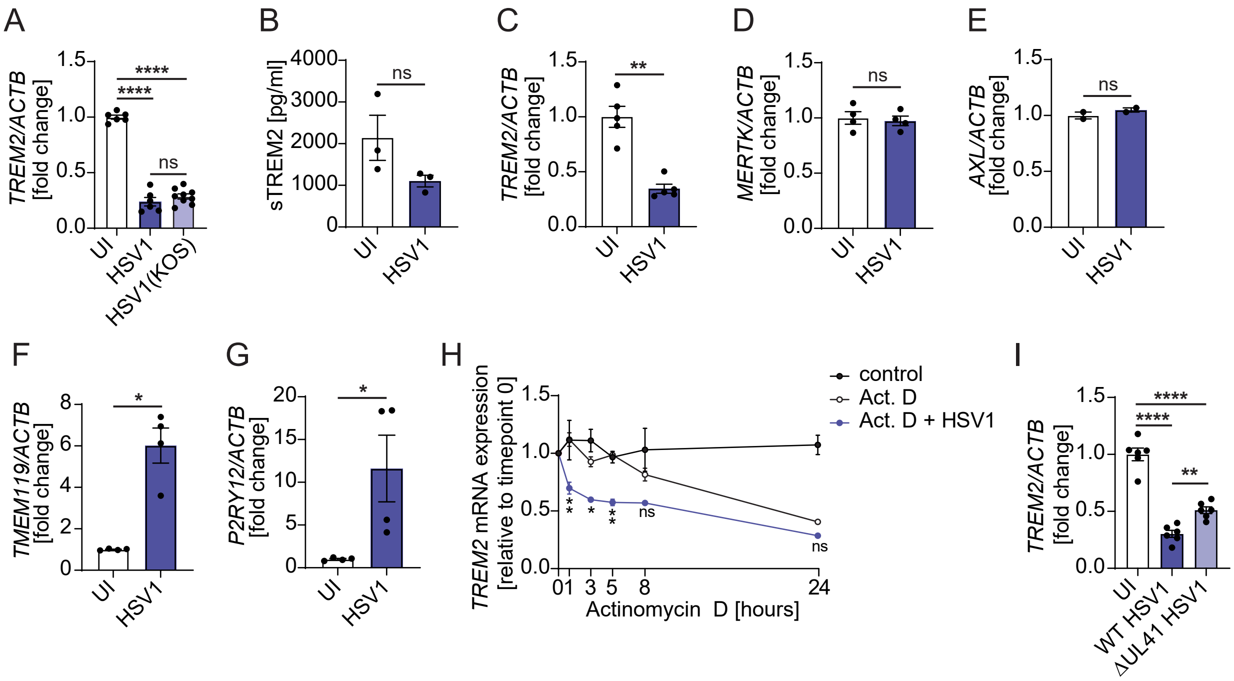

Fig. S2. HSV1 downregulates microglial TREM2 expression independent of the viral strain and iPSC line used. (**A**) TREM2 mRNA levels were analyzed 24 h post infection of hiPSC-derived microglia with the clinical HSV1 strain (MOI 1) and the KOS strain (MOI 1). (B, C) Microglia differentiated from a separate iPSC line (ChiPSC22). (**B**) sTREM2 levels were analyzed 24 h post infection with HSV1 (MOI 1) by ELISA. (**C**) TREM2 mRNA levels were analyzed 24 h post infection with HSV1 (MOI 1). (**D-G**) Examples of genes which mRNA levels were unchanged (MERTK and AXL) or upregulated (TMEM119 and P2RY12) in microglia 24 h post infection with HSV1 (MOI 1). (**H**) Microglia were incubated with vehicle control or 5 µg/ml Actinomycin D for the indicated time points in presence and absence of HSV1 infection (MOI 3). TREM2 mRNA decay was quantified. (**I**) TREM2 mRNA levels were analyzed 24 h post infection with wildtype (WT) HSV1 and a UL41 mutant strain (ΔUL41). All figures represent 2-3 independent experiments; data are presented as mean ± SEM; P values were calculated by one-way ANOVA with Tukey's multiple comparisons test (A and I), Mann-Whitney U test (B-G) and two-way ANOVA with Tukey's multiple comparisons test (H). * $P<0.05$; ** $P<0.001$; **** $P<0.0001$.

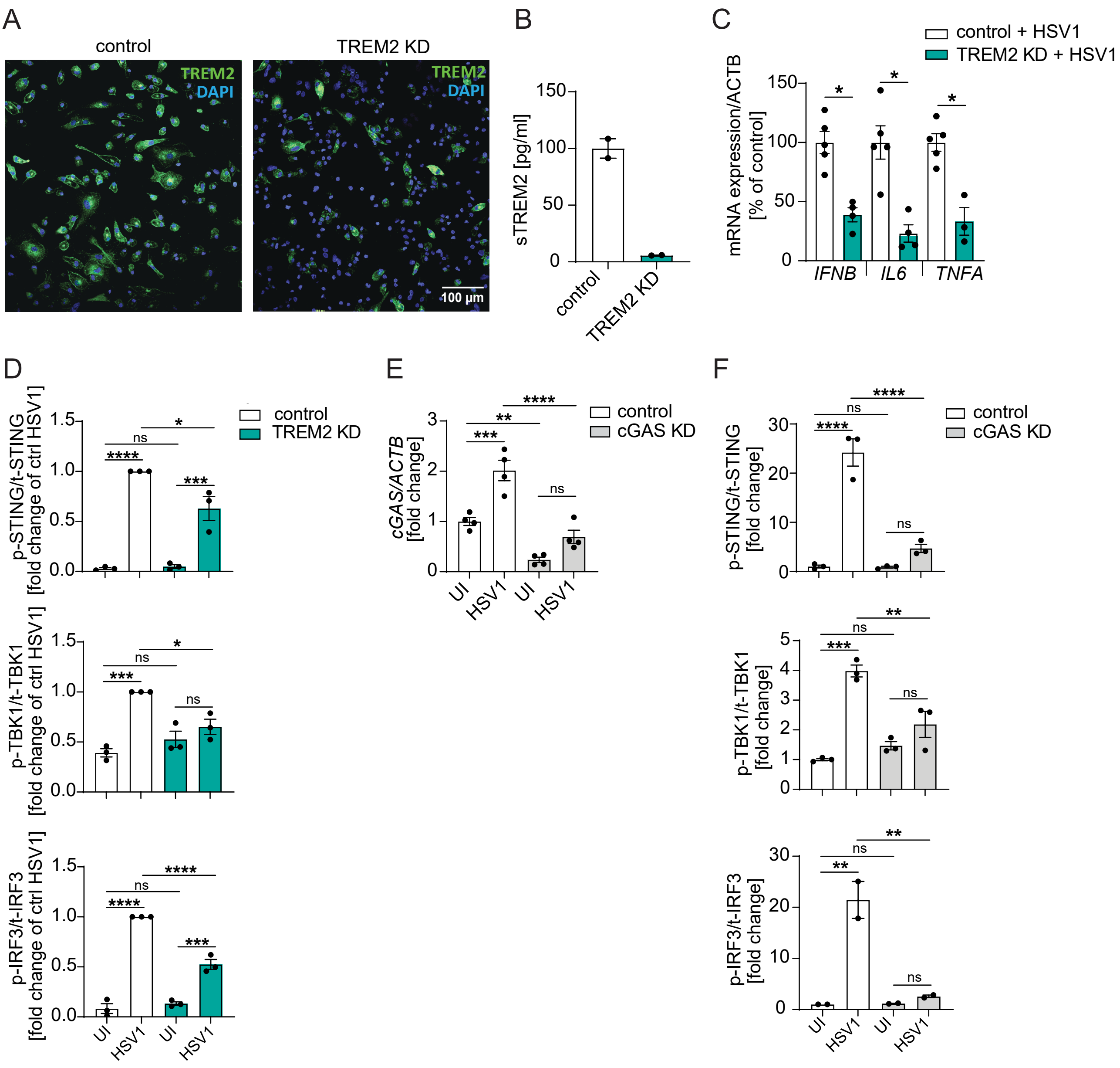

Fig. S3. Characterization of TREM2 KD hiPSC-derived microglia. (**A**) Control and TREM2 KD microglia were stained for TREM2 (green) and DAPI (blue) and imaged using a confocal microscope. (**B**) sTREM2 levels were measured in cell supernatants by ELISA. (**C**) Microglia differentiated from a separate iPSC line (ChiPSC22). IFNB, IL6, and TNFA mRNA levels were analyzed 24 h post infection with HSV1 (MOI 3). (**D**) Quantifications of Fig. 3F (**E**) cGAS was knocked down in microglia using siRNA. Cells were used for experiments 4 days after transfection. Control and cGAS KD microglia were infected with HSV1 (MOI 3) for 24 h. cGAS mRNA levels were quantified. (**F**) Quantifications of Fig. 2K. All figures represent 2-3 independent experiments; data are presented as mean ± SEM; P values were calculated by Mann-Whitney U test (B,C) and one-way ANOVA with Tukey's multiple comparisons test (D-F). * P<0.05; ** P<0.001; *** P<0.0005; **** P<0.0001.

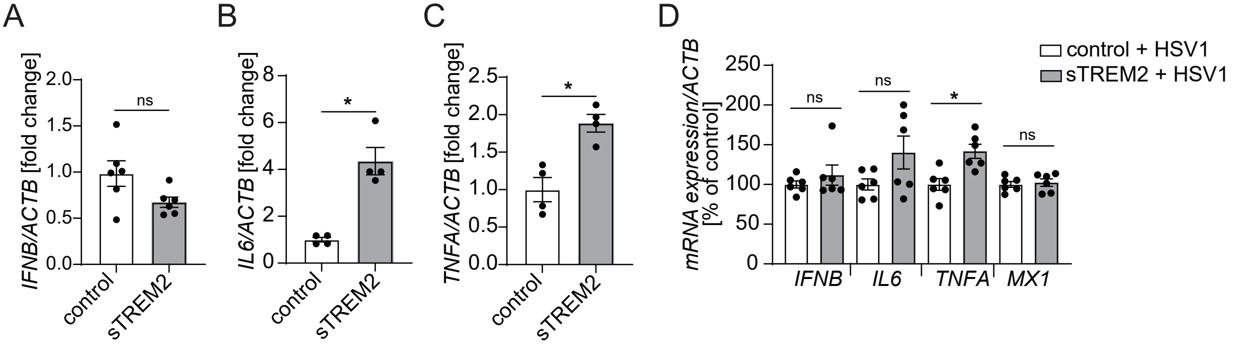

Fig. S4. sTREM2 activates inflammatory responses but not antiviral defense. (**A-C**) HiPSC-derived microglia were incubated with 3 µg/ml sTREM2 for 24 h. IFNB, IL6, and TNFA mRNA levels are shown. (**D**) IFNB, IL6, TNFA, and MX1 mRNA levels were analyzed in microglia after infection with HSV1 (MOI 1) with and without sTREM2 for 8 h. All figures represent 2-3 independent experiments; data are presented as mean ± SEM; P values were calculated by Mann-Whitney test. * P<0.05.

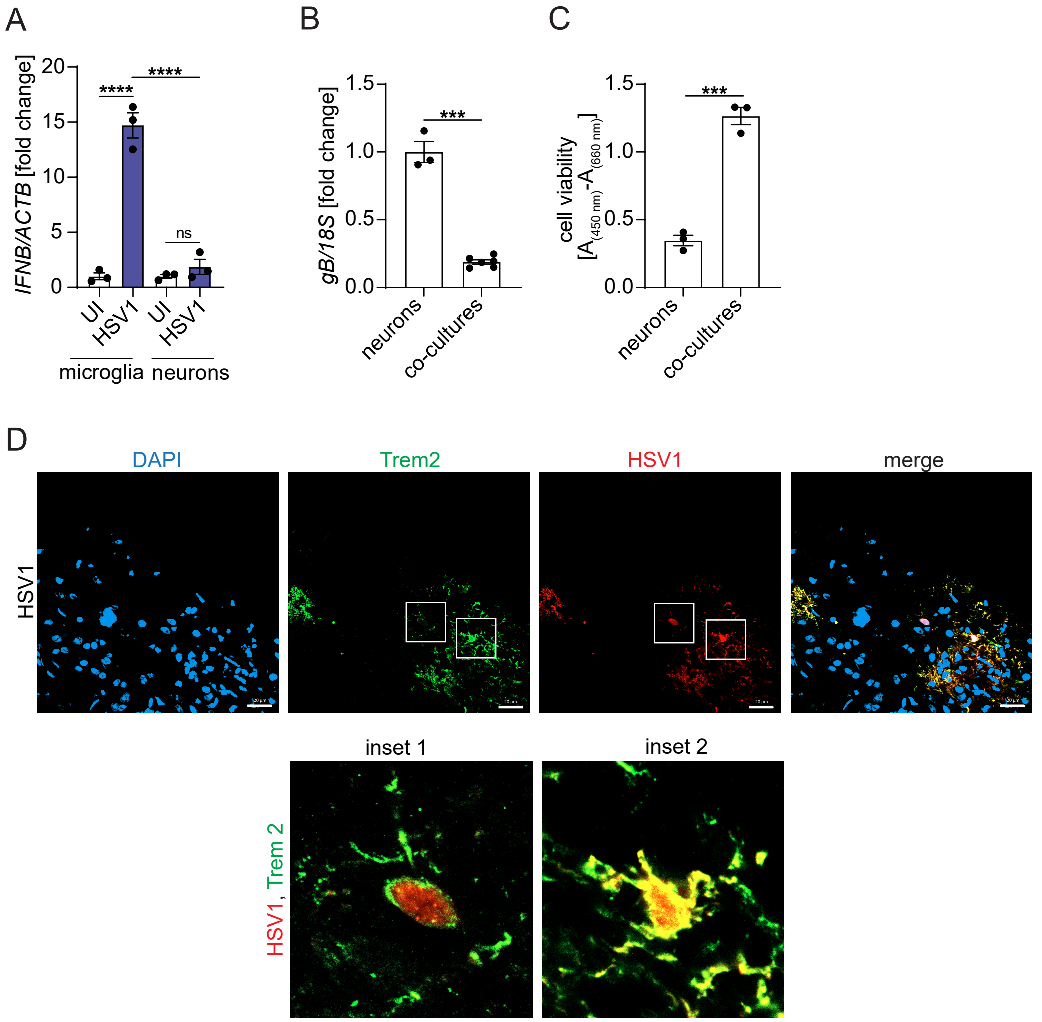

Fig. S5. Microglia are the main source of IFNB and decrease viral replication in co-cultures compared with neurons alone. (**A**) IFNB mRNA levels were analyzed in hiPSC-derived microglia and neurons 24 h after infection with HSV1 (MOI 1). (**B**, **C**) HiPSC-derived neurons and co-cultures of microglia and neurons were analyzed 24 h after infection with HSV1 (MOI 1). (**B**) gB mRNA levels are shown. (**C**) Cell viability is shown. (**D**) WT mice were infected with HSV1 (2 × 106 PFU/cornea), brainstems were isolated and stained 5 days post infection. Tissue sections were stained for HSV1 (red) and Trem2 (green). Representative images are shown. Scale bar = 20 µm. Two insets are magnified without DAPI. All figures represent 1-2 independent experiments; data are presented as mean ± SEM; P values were calculated by two-way ANOVA with Tukey's multiple comparisons test (A) and two-tailed Student's t-test (B, C). *** P<0.0005; **** P<0.0001.

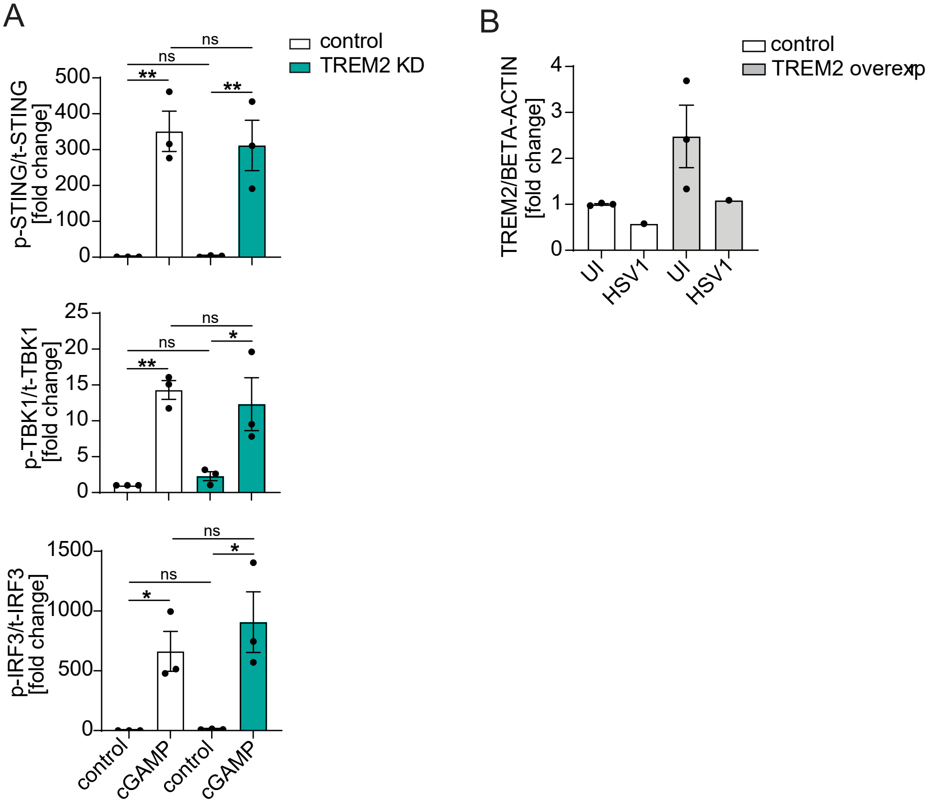

Fig. S6. Quantification of cGAS/STING signaling in cGAMP-stimulated microglia. (**A**) Quantifications of Fig. 5B are shown. (**B**) Quantification of Fig. 6C. (**A**) represents three independent experiments; (**B**) represents one experiment. Data are presented as mean ± SEM; P values were calculated by one-way ANOVA with Tukey's multiple comparisons test. * P<0.05; ** P<0.001.

Data S1. List of DEGs in HSV1-infected microglia compared to controls as identified by RNAseq.

Data S2. List of the 200 most downregulated genes in HSV1-infected microglia compared to controls as identified by RNAseq.
